# Development of an individualized model for predicting postoperative delirium in elderly patients with hepatocellular carcinoma

**DOI:** 10.1038/s41598-024-62593-z

**Published:** 2024-05-22

**Authors:** Mingfang Yan, Zhaoyan Lin, Huizhe Zheng, Jinglan Lai, Yuming Liu, Zhenmeng Lin

**Affiliations:** 1 Department of Anesthesiology, Clinical Oncology School of Fujian Medical University &, Fujian Cancer Hospital, Fuzhou, 350014 Fujian China; 2 College of Animal Science, Fujian Agriculture and Forestry University, Fuzhou, 350002 Fujian China; 3 Department of Infectious Diseases, Mengchao Hepatobiliary Hospital of Fujian. Medical University, Fuzhou, 350025 Fujian China; 4 Department of Anesthesiology, Mengchao Hepatobiliary Hospital of Fujian. Medical University, Fuzhou, 350025 Fujian China

**Keywords:** Cancer, Diseases, Medical research, Oncology, Risk factors, Signs and symptoms

## Abstract

Postoperative delirium (POD) is a common complication in older patients with hepatocellular carcinoma (HCC) that adversely impacts clinical outcomes. We aimed to evaluate the risk factors for POD and to construct a predictive nomogram. Data for a total of 1481 older patients (training set: n=1109; validation set: n=372) who received liver resection for HCC were retrospectively retrieved from two prospective databases. The receiver operating characteristic (ROC) curve, calibration plot, and decision curve analysis (DCA) were used to evaluate the performance. The rate of POD was 13.3% (148/1109) in the training set and 16.4% (61/372) in the validation set. Multivariate analysis of the training set revealed that factors including age, history of cerebrovascular disease, American Society of Anesthesiologists (ASA) classification, albumin level, and surgical approach had significant effects on POD. The area under the ROC curves (AUC) for the nomogram, incorporating the aforementioned predictors, was 0.798 (95% CI 0.752–0.843) and 0.808 (95% CI 0.754–0.861) for the training and validation sets, respectively. The calibration curves of both sets showed a degree of agreement between the nomogram and the actual probability. DCA demonstrated that the newly established nomogram was highly effective for clinical decision-making. We developed and validated a nomogram with high sensitivity to assist clinicians in estimating the individual risk of POD in older patients with HCC.

Hepatocellular carcinoma (HCC) ranks as the sixth most commonly diagnosed cancer and the third deadliest globally^1^. Globally, the incidence rates of HCC exhibit a positive correlation with age, reaching their peak around the age of 75 years^2,3^. Owing to rapid aging of the global population and a record-high average life expectancy, there is a growing incidence of older patients diagnosed with HCC^4,5^.

Postoperative delirium (POD) is a prevalent and serious complication marked by acute and varying alterations in mental condition, attentional capabilities, and consciousness levels following liver resection^6–8^. Studies have shown a correlation between POD and unfavorable consequences, including heightened mortality rates, extended hospital stays, and elevated medical costs. Additionally, POD may contribute to lasting and more substantial declines in cognitive functions and daily life activities^9–13^.

Roughly one-third of POD cases are considered preventable, making it a suitable focus for surgical quality improvement endeavors^14,15^. In practice, uniformly implementing all effective delirium prevention strategies for every older surgical patient throughout their perioperative course is often impractical, despite being theoretically possible. Given the resource constraints and infrequent implementation of these interventions in most centers, recommendations have been made to focus on identifying patients with the highest risk^16–18^.

Previous research has established nomograms for POD in malignant tumors, including gynecologic cancers^19^, gastric cancer ^20^, colorectal cancer^21^, and head and neck cancer^22^. However, the accuracy of these nomograms varies widely and may not necessarily be applicable to HCC. In this study, we aimed to identify the risk factors for POD in older patients with HCC and to develop a corresponding nomogram.

## Materials and methods

### Patients

**Figure 1:**
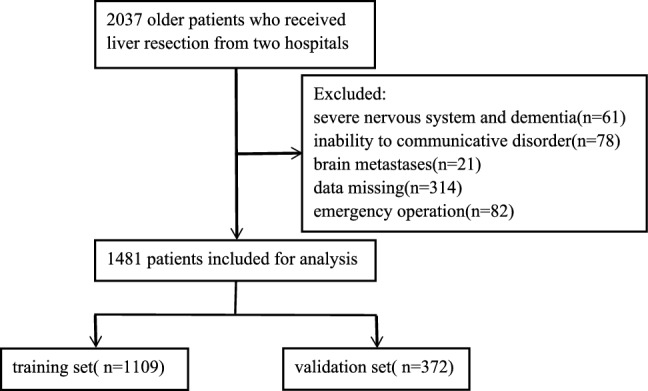
Flowchart of patient selection.

A nomogram was developed through a retrospective analysis of a prospectively registered database including 1109 patients with HCC at Mengchao Hepatobiliary Hospital of Fujian Medical University between March 2015 and June 2020. Concurrently, for external validation, we included data from 372 patients treated at Fujian Cancer Hospital between March 2018 and August 2020 (Fig. 1). The inclusion criteria were: (1) individuals aged 65 years and older; (2) patients who underwent elective hepatectomy; and (3) availability of sufficient data. The exclusion criteria were: (1) preoperative cognitive impairment; (2) history of severe nervous system disorders and dementia; (3) language barriers; hearing or vision impairments resulting in an inability to communicate; and (4) brain metastases.

This study was performed in accordance with the Declaration of Helsinki. Written informed consent was obtained from all participants. This research was approved by the ethics committee of Mengchao Hepatobiliary Hospital of Fujian Medical University.

### Definitions

The diagnosis of POD was conducted through the application of the Confusion Assessment Method (CAM), a widely used diagnostic algorithm known for its demonstrated high sensitivity and specificity in identifying delirium^23,24^. POD frequently initiates in the recovery room and can persist for up to 5 days following the surgical procedure^18^. Assessment of POD can occur daily for a consecutive 5-day period post surgery, whenever patients manifest an abrupt alteration in mental status.

Cerebrovascular disease, as defined in this study, included conditions such as cerebral infarction, cerebral hemorrhage, stenosis (such as stenosis of the carotid, vertebral stenosis, or intracranial stenosis), and aneurysms^25^.

Ischemic heart disease (IHD) refers to inadequate blood supply to the heart, resulting in myocardial ischemia. This encompasses acute myocardial infarction, chronic stable angina, chronic IHD, and its associated heart failure^25,26^.

Chronic pulmonary disease is characterized by the presence of at least one of the following conditions: asthma, chronic obstructive pulmonary disease, and restrictive lung disease^27^.

Intraoperative hypotension is characterized by instances in which the patient experiences a systolic blood pressure below 80 mmHg or encounters at least one episode of systolic blood pressure that falls more than 20% below the baseline^28^.

### Statistical analysis

The data were analyzed using both IBM SPSS 24.0 (IBM Corp) and R software (version 4.1.1). Parameters with a normal distribution are expressed as mean ± standard deviation and analyzed using the Student *t*-test. Parameters not following a normal distribution are expressed as median and interquartile range and analyzed using the Mann–Whitney test. Categorical variables are presented as frequency and percentage, and their comparisons were conducted using the chi-squared test. The least absolute shrinkage and selection operator (LASSO) regression model was used to select the optimal predictive variables. Subsequently, the identified key features were integrated into multivariable logistic regression analysis. Forest plots, constructed using GraphPad Prism, were used to visualize the results. Predictors found to be statistically significant were used to establish a nomogram system for diagnosing POD. The discriminative performance of the nomogram was assessed using the area under the receiver operating characteristic curve (AUC). Calibration curves were generated to evaluate the concordance between predicted and observed probabilities. Additionally, a decision curve analysis (DCA) was conducted to assess the clinical utility of the nomogram. Statistical significance was defined as a two-tailed P-value < 0.05.

## Results

### Patient characteristics

In the training set, the overall rate of POD was 13.3% (148 of 1109 patients). In the validation set, this rate was slightly higher at 16.4% (61 of 372 patients). Notable differences between the two sets included factors such as sex, body mass index, presence of hypertension, history of previous abdominal surgery, platelet levels, blood urea nitrogen, duration of the surgical procedure, and a requirement for intraoperative blood transfusion (Table 1).

**Table 1:** Patients’ background characteristics in the training and validation sets.

Variables	Training cohort (n = 1109)	Validation cohort (n = 372)	P-value
Age, years, mean (SD)	70.1 ± 4.9	69.8 ± 4.6	0.283
Gender, n (%)			0.023
Male	816	251	
Female	293	121	
Education, n (%)			0.692
< High school	928	308	
≥ High school	181	64	
Marital status, n (%)			0.132
Married	745	234	
Spinsterhood/Divorced/widowed	364	138	
BMI, kg/m^2^, mean (SD)	22.2 ± 2.2	22.7 ± 2.4	< 0.001
History, n (%)			
Hypertension	386	100	0.005
Diabetes mellitus	298	107	0.479
Ischemic heart disease	199	74	0.402
Cerebrovascular disease	108	44	0.252
Liver cirrhosis	290	85	0.205
Arrhythmia	220	87	0.144
Psychiatric disorder	90	41	0.088
Pulmonary disease	269	98	0.420
Previous malignancy	177	67	0.356
Previous abdominal surgery	127	62	0.009
Smoking, n (%)	306	95	0.440
Drinking, n (%)	390	122	0.405
Preoperative medications, n (%)			
Sleeping pills	105	39	0.567
Statins	257	85	0.898
ASA classification, n (%)			0.504
I-II	858	294	
III-IV	251	78	
FEV1/FVC, %, mean (SD)	84.8 ± 7.5	84.3 ± 7.1	0.233
Preoperative blood test			
WBC, × 10^9^/L, mean (SD)	6.7 ± 1.9	6.5 ± 1.8	0.198
Platelets, × 10^9^/L, mean (SD)	244.0 ± 77.9	256.8 ± 76.7	0.006
Hemoglobin, g/L, mean (SD)	126.7 ± 18.4	124.7 ± 16.9	0.062
BUN, mmol/L, mean (SD)	6.3 ± 1.2	6.5 ± 1.1	0.015
Creatinine, μmol/L, mean (SD)	87.9 ± 13.7	86.7 ± 13.8	0.142
Total bilirubin, μmol/L, mean (SD)	13.7 ± 4.2	13.2 ± 4.4	0.075
ALT, U/L, mean (SD)	22.9 ± 8.6	20.8 ± 6.7	0.055
AST, U/L, mean (SD)	25.8 ± 10.2	22.4 ± 6.1	0.052
Albumin level, g/L, mean (SD)	38.1 ± 6.4	37.5 ± 6.1	0.088
Prothrombin time, s, median(IQR)	11.8(11.5, 12.8)	11.7(11.3, 12.9)	0.260
Child Pugh classification			0.505
A	1045	347	
B	64	25	
Largest tumor size, mm, median (IQR)	4.1(3.1, 6.3)	4.4(3.2, 7.1)	0.098
Tumor number			0.444
Single	888	291	
Multiple	221	81	
Duration of procedure, min, mean (SD)	274.3 ± 63.5	257.8 ± 64.3	< 0.001
Intraoperative blood loss, ml, median, (IQR)	310(210, 420)	330(220, 430)	0.240
Surgical approach, n (%)			0.194
Minimally invasive	502	154	
Open	607	218	
Extent of resection			0.265
Wedge resection, n (%)	406	135	
Segmentectomy, n (%)	242	69	
Sectionectomy, n (%)	262	105	
Resection of ≥ 2 sections, n (%)	199	63	
Intraoperative hypotension, n (%)	203	75	0.427
Intraoperative blood transfusion,n (%)	257	59	0.003

*ASA* American society of anesthesiologists, *BUN* Blood urea nitrogen, *BMI* Body mass index, *IQR* Interquartile range, *SD* Standard deviation, *ALT* Alanine transaminase, *AST* Aspartate transaminase, *FEV1/FVC* Ratio of forced expiratory volume in the first 1 s to forced vital capacity.

### Selection of independent predictors for POD and development of the nomogram in the training set

**Figure 2:**
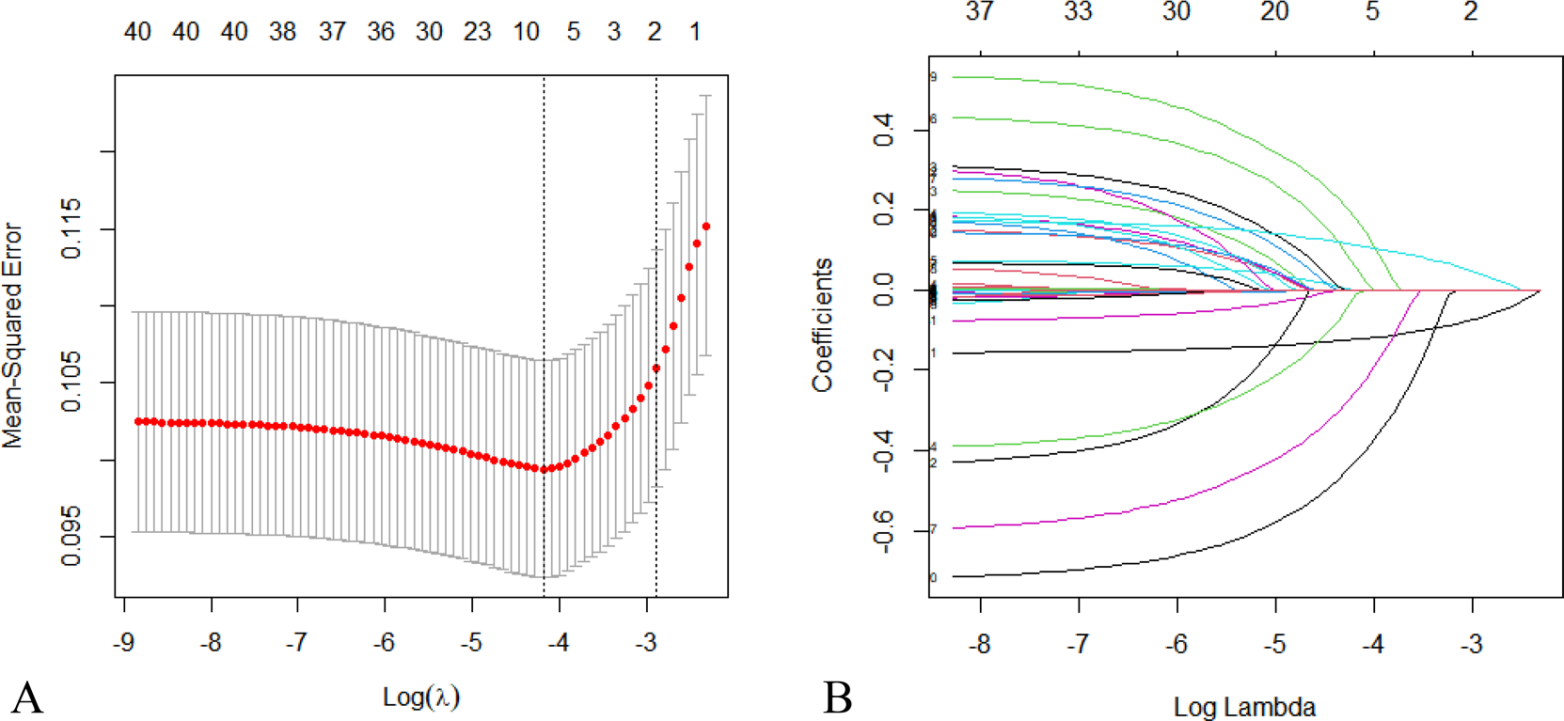
Clinicopathologic feature selection using the LASSO regression model. (**A**) The smallest lambda is determined through tenfold cross-validation. (**B**) LASSO coefficient profiles of 40 signatures. When the smallest lambda equals 0.015, the eight coefficients with non-zero values are selected.

**Figure 3:**
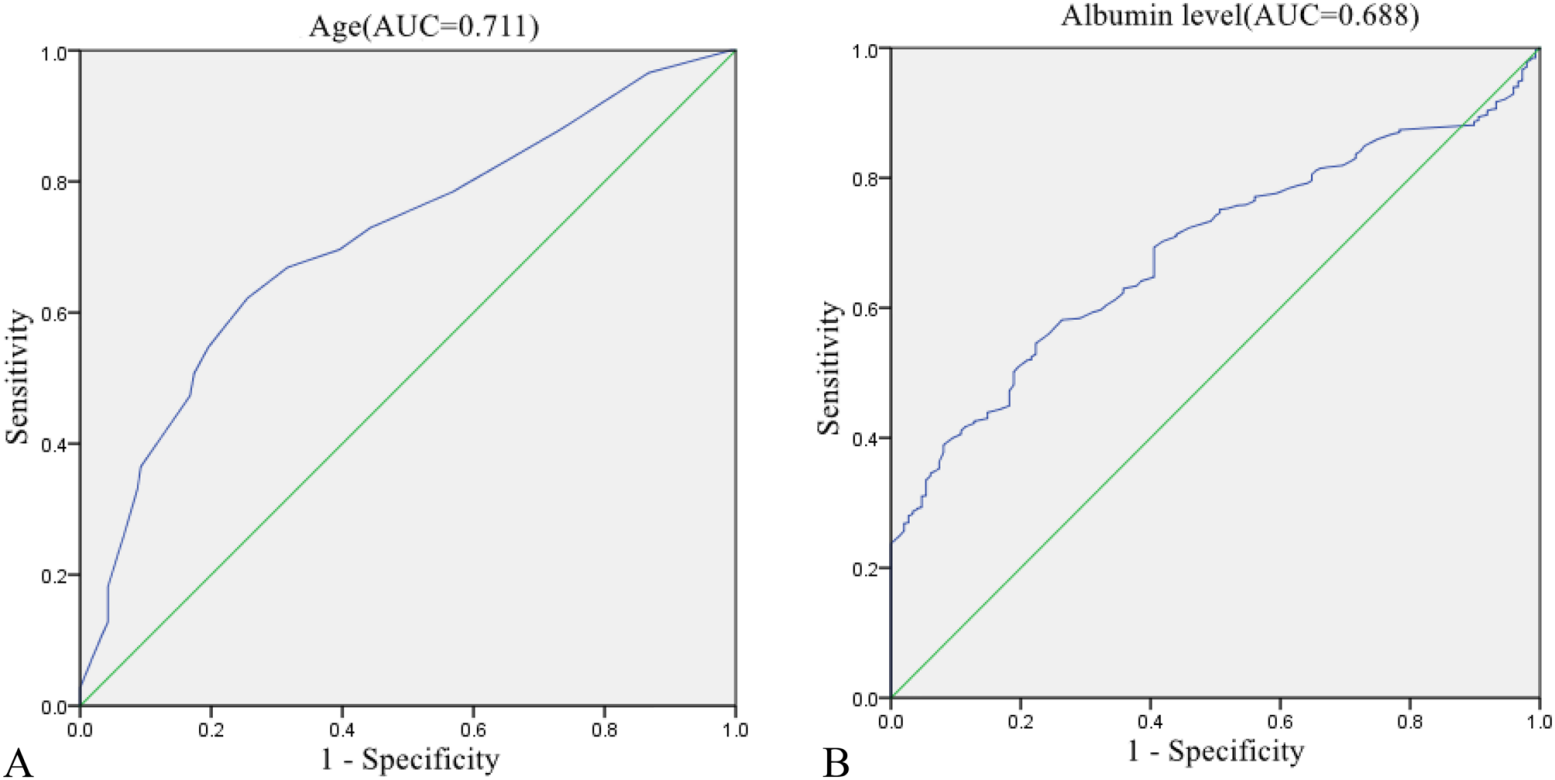
Receiver operating characteristic (ROC) curves of the age (**A**) and albumin level (**B**). *AUC* Area under the ROC curve.

**Figure 4:**
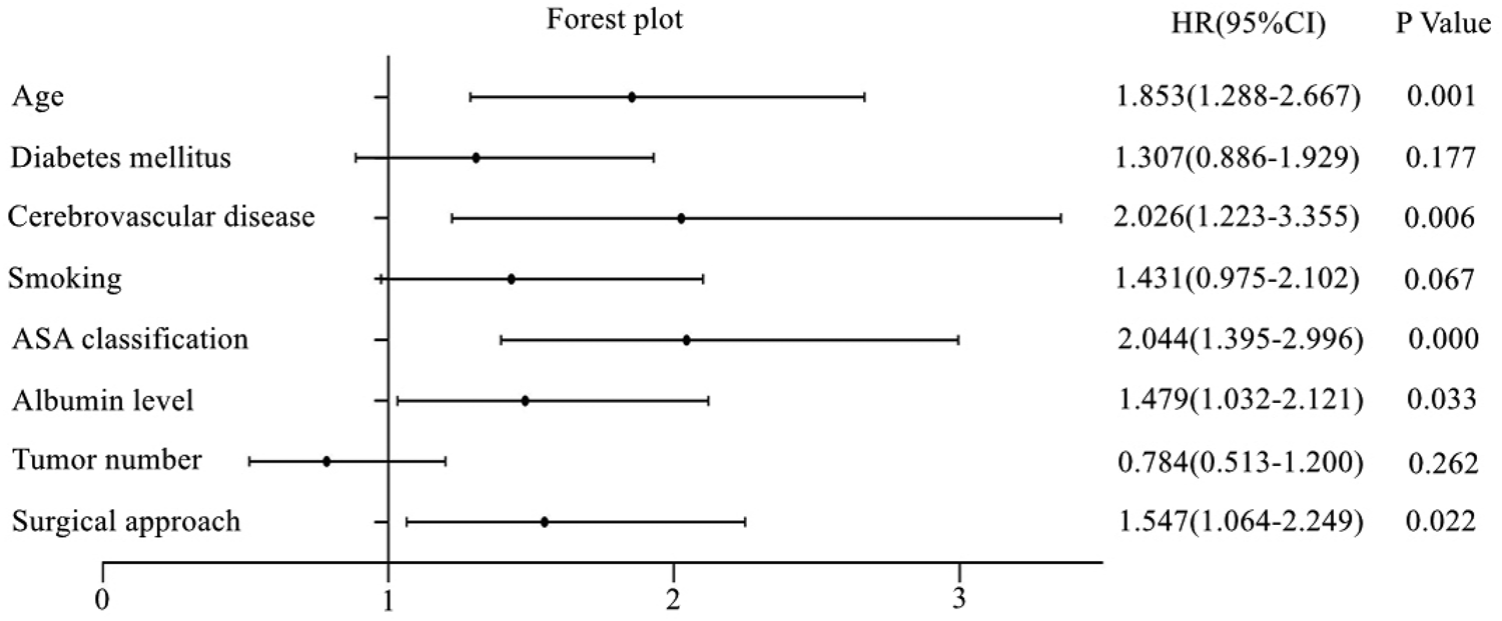
Forest plot of independent predictors of postoperative delirium. *ASA* American Society of Anesthesiologists, *HR* Hazard ratio, *CI* Confidence interval.

From a pool of 40 related dependent variables, eight potential predictors with non-zero coefficients in the LASSO regression model were selected based on data from the training set. These predictors include age, diabetes mellitus, history of cerebrovascular disease, smoking, ASA classification, albumin level, tumor number, and surgical approach (Fig. 2). To facilitate analysis and clinical implementation, continuous variables were dichotomized according to the ROC curve analysis. The optimal cutoff values for age and albumin level were determined to be 71 (AUC = 0.711) and 37.2 g/L (AUC = 0.688), respectively (Fig. 3). The multivariable logistic regression analysis incorporated these predictors, with age, history of cerebrovascular disease, ASA classification, albumin level, and surgical approach recognized as independent risk factors for POD (Fig. 4).

### Construction and validation of the nomogram

**Figure 5:**
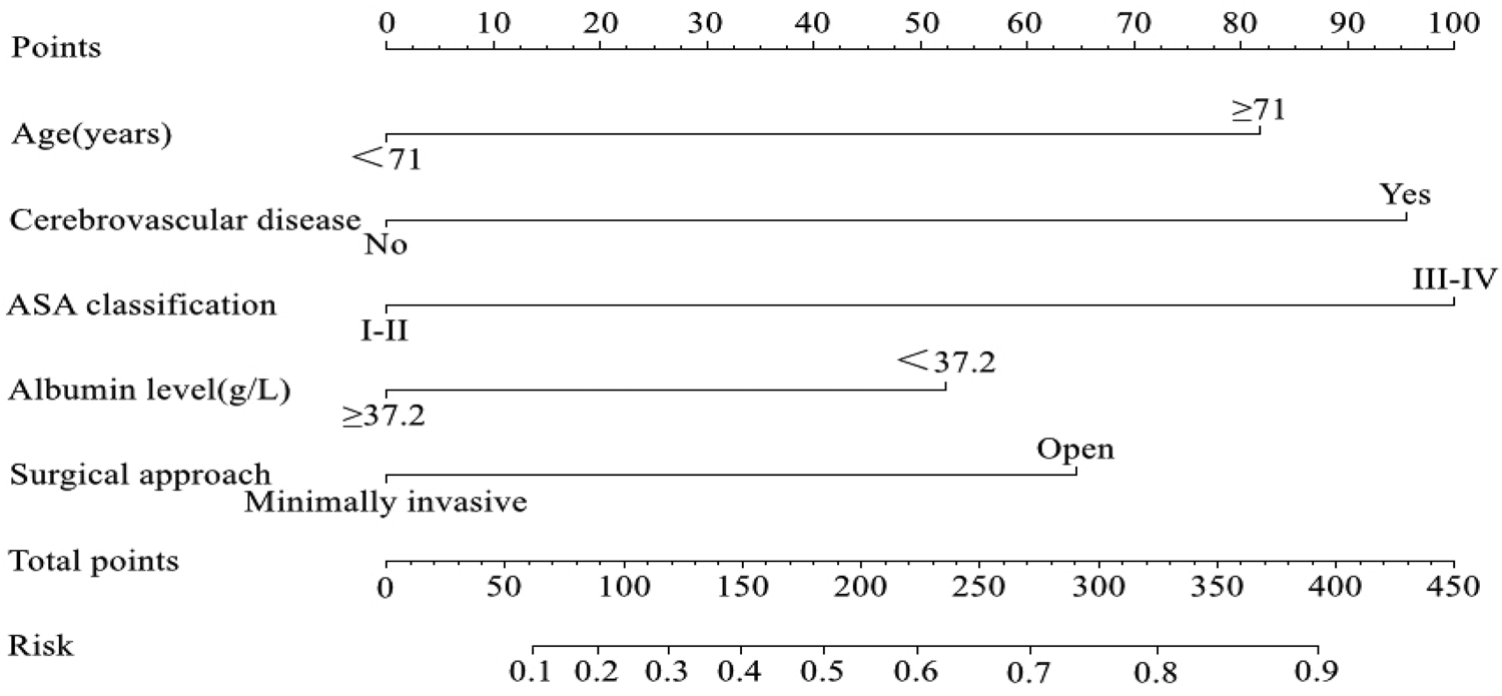
Nomogram for estimating the likelihood of POD in older patients diagnosed with HCC. *ASA* American Society of Anesthesiologists.

**Figure 6:**
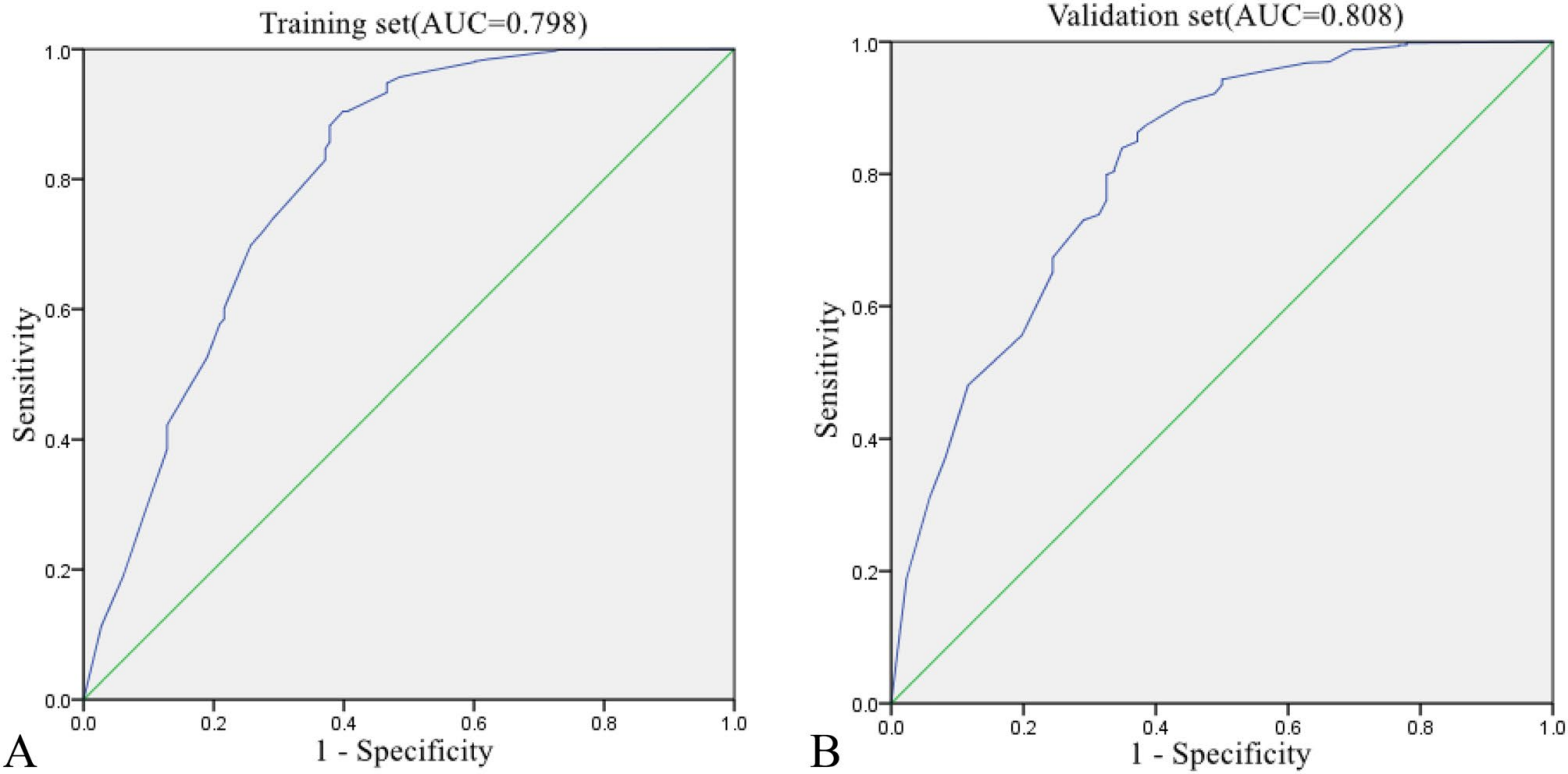
Receiver operating characteristic (ROC) curves in the training set (**A**) and validation set (**B**). *AUC* Area under the ROC curve.

**Figure 7:**
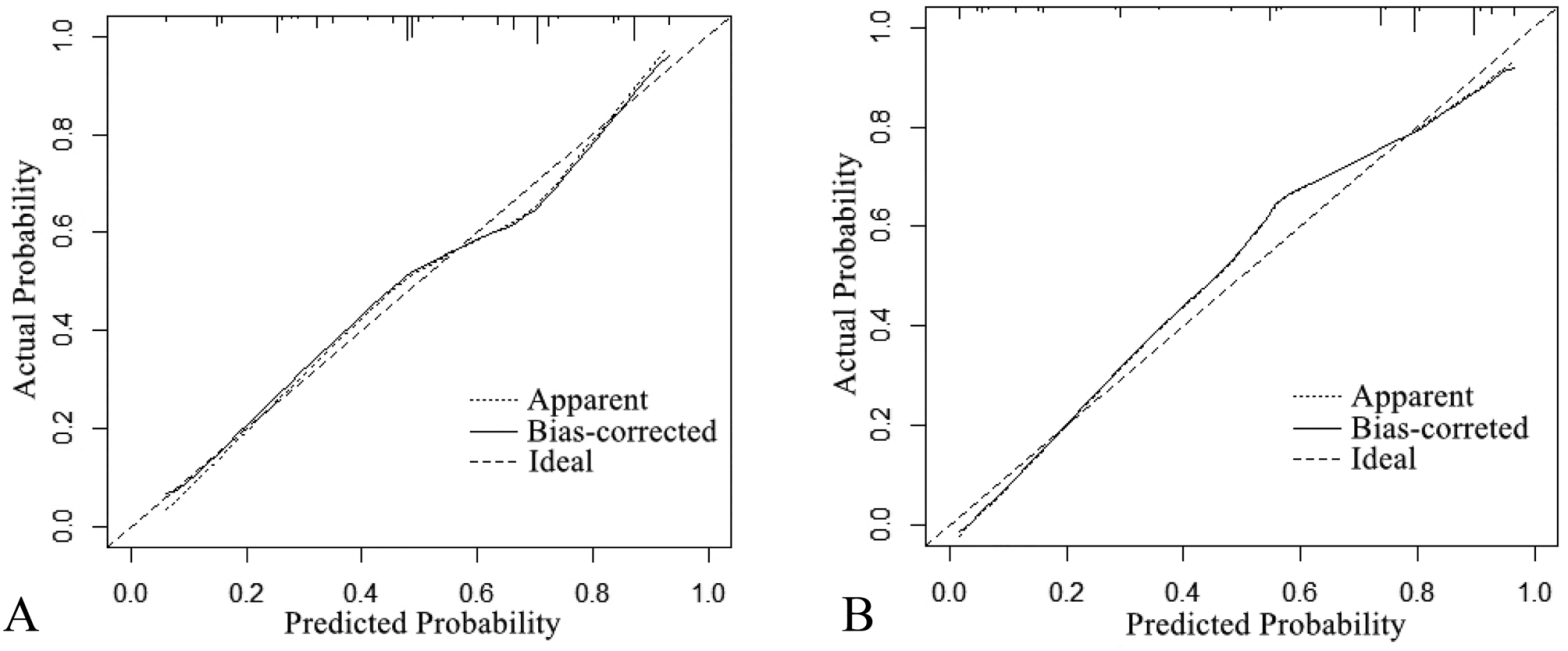
Calibration curves of the nomogram in the training set (**A**) and validation set (**B**). The horizontal axis depicts the anticipated likelihood of POD, and the vertical axis illustrates the actual occurrence of diagnosed POD relative to the total cases. The diagonal dashed line represents the perfect prediction of the ideal model. The solid line represents the prediction of the nomogram; a closer fit to the diagonal dashed line represents the result after bias correction by bootstrapping (1000 repetitions).

**Figure 8:**
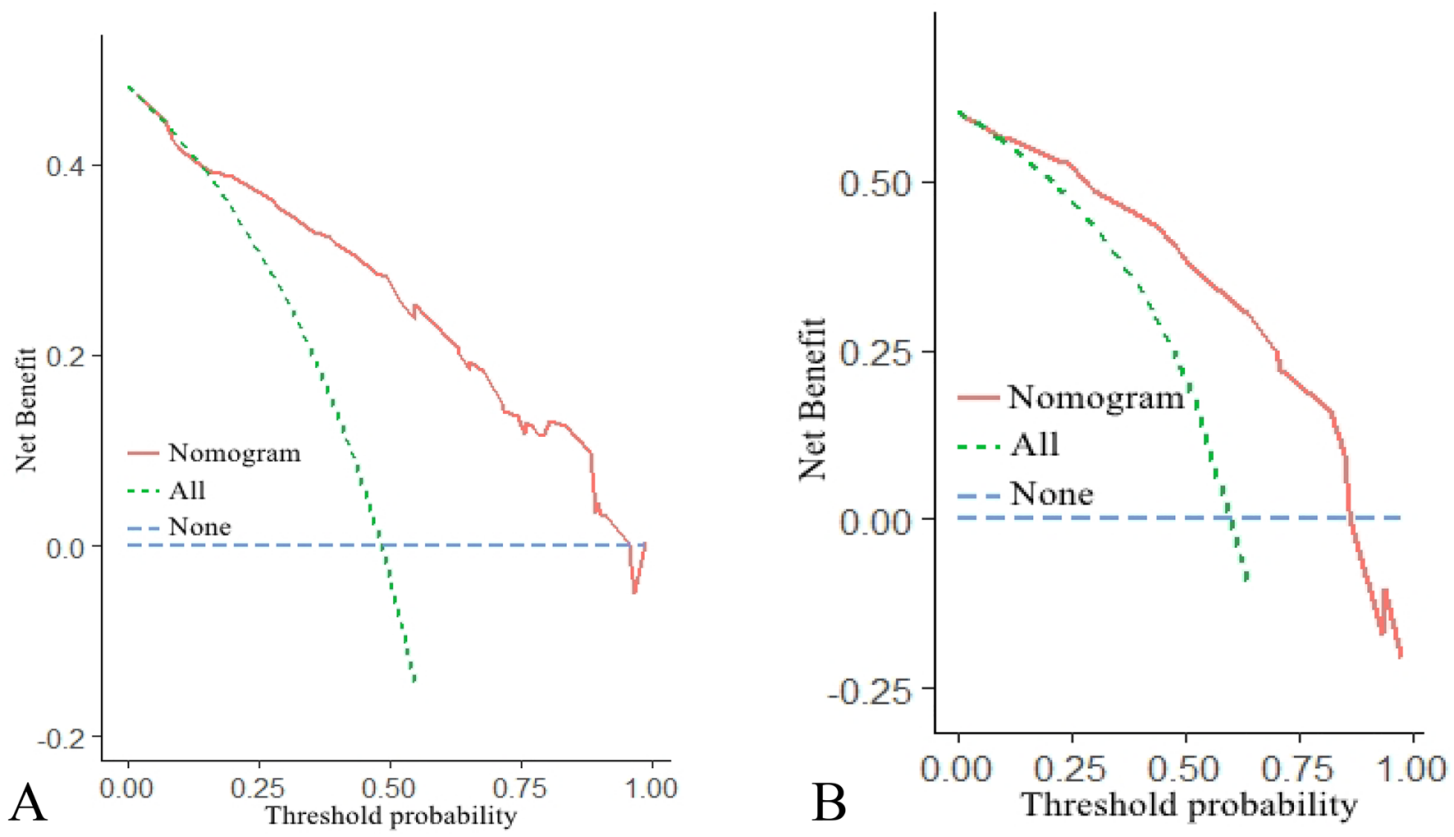
Decision curve analysis of the nomogram in the training set (**A**) and validation set (**B**). The net benefit is quantified along the y-axis. The red line denotes predictions from the nomogram, the green line signifies the assumption of POD occurrence in all patients, and the blue line signifies the assumption of no POD occurrence in any patient.

The nomogram was created using these independent factors as its foundation (Fig. 5). The AUCs of the nomogram model were 0.798 (95% CI 0.752–0.843) in the training set and 0.808 (95% CI 0.754–0.861) in the validation set (Fig. 6). The calibration curves generated by the nomogram exhibited strong concordance between observed outcome frequencies and predicted probabilities in both sets (Fig. 7). The results of DCA are shown in Fig. 8, which demonstrated that the nomogram used in our study had superior effectiveness compared with treating all patients or providing no treatment. This advantage was observed when the threshold probability ranged from 9 to 91% in the training set and from 5 to 87% in the validation set.

## Discussion

The incidence of POD was 13.3% in the training set and 16.1% in the validation set. Consistent with prior research, Nomi et al. reported a POD rate of 14.2%^29^. Yoshimura et al. found a POD rate of 17.0% in patients undergoing hepatectomy^30^. However, Ishihara et al. reported a lower POD incidence of 7.5%^6^, and Chen et al. found this to be 8.4%^31^. Variability in POD rates may be attributed to several factors: a lack of consistent definitions and assessment methods for POD by researchers, the diverse clinical characteristics of patients, and inaccuracies in estimation owing to the use of retrospective research methods.

This research involved developing and validating a new nomogram for predicting POD in older HCC patients. The model demonstrated strong capabilities in both discrimination and calibration, which showed its clinical value. The model's robustness was further enhanced by external validation, affirming its applicability across different patient groups and clinical settings. To our knowledge, this represents the inaugural predictive model for POD in individuals diagnosed with HCC.

Previous studies have indicated a correlation between the risk of POD and the emergence of postoperative complications^32,33^. However, such data are not accessible before or during surgery and thus cannot be integrated into predictive models.

In this study, a significant correlation between advanced age and an increased risk of POD was observed. Older patients experience a decline in physical capabilities, brain tissue integrity, and stress response regulation, along with diminished levels of key central neurotransmitters like acetylcholine and epinephrine^34^. Age-related constriction of blood vessels reduces cerebral oxygenation, which can potentially lead to postoperative cerebral impairment^35,36^. Moreover, alterations in drug metabolism and response owing to aging may increase the adverse effects of medications, thereby increasing the likelihood of POD^37^.

The present study identified that a history of cerebrovascular disease is an independent risk factor for POD. Cerebrovascular disease can lead to cognitive impairment, dementia, and neurocognitive deficits, which is postulated to increase delirium possibly through altered brain networks and a reduced ability to integrate sensory inputs^38^. Long-term susceptibility to delirium should be regarded as an integral aspect of the overall cerebrovascular disease burden^39,40^. Several studies have indicated that cognitive dysfunction and reduced functional capacity are associated with a heightened risk of POD^41–43^.

The ASA physical status classification system is commonly applied to evaluate a patient's ability to withstand anesthesia, primarily based on their overall compromised health and the presence of multiple comorbidities^44^. Research has indicated that an ASA classification ≥ 3 is associated with an increased risk of complications and decreased overall survival after hepatectomy^45–47^. Our study indicated that an ASA classification ≥ 3 is a risk factor for POD, as evidenced in numerous studies on this topic^48–51^. Whereas we found no statistically significant differences in common comorbidities such as diabetes and hypertension between the groups, it is considered that the cumulative impact of various comorbidities might heighten baseline vulnerability in older patients. This susceptibility, combined with the stress of surgery, could be a contributing factor to the development of POD^52,53^.

Numerous research findings indicate that a lower patient albumin level increases their likelihood of experiencing POD, a conclusion that aligns with the findings of our study^6,54–57^. Hypoalbuminemia affects drug metabolism, antioxidant defense, and toxin processing because albumin is the primary transport protein in blood plasma. Reduced albumin levels may result in cognitive dysfunction owing to toxic effects and oxidative injuries^58,59^. Appropriate medical intervention can yield lower albumin levels, potentially aiding in the reduction of POD risk.

This study showed that an open approach independently increases the risk of POD. A laparoscopic approach may reduce operative stress and postoperative systemic inflammation, which are known to be linked to the occurrence of POD^29,60–62^.

There are a number of limitations in this study. First, this research was a retrospective evaluation conducted using a prospectively registered database. Recognizing the intrinsic biases inherent in this type of study design is crucial. Prior studies have highlighted several risk factors linked to POD, including preoperative depression and anxiety^63–68^. Nevertheless, these factors were not incorporated into our analysis owing to certain constraints. Second, the experiment was conducted in only two centers, both of which are located in the same city. To further validate the model, it is necessary to use a more extensive sample size and conduct studies across various centers in different regions.

## Conclusion

In older patients with HCC, factors such as age, cerebrovascular disease history, ASA classification, albumin levels, and the type of surgical procedure are identified as independent predictors of POD. In this study, we developed and externally validated a new, precise nomogram for personalized assessment and clinical decision-making.

## Data Availability

The datasets generated during and/or analyzed during the current study are available from the corresponding author on reasonable request.

## References

[1] Sung H, Ferlay J, Siegel RL, Laversanne M, Soerjomataram I, Jemal A, Bray F (2021). Global cancer statistics 2020: GLOBOCAN estimates of incidence and mortality worldwide for 36 cancers in 185 countries. CA Cancer J. Clin..

[2] Xu L, Xu Y, Li G, Yang B (2022). Perioperative factors related to the prognosis of elderly patients with hepatocellular carcinoma. Eur. J. Med. Res..

[3] Petrick JL (2020). International trends in hepatocellular carcinoma incidence, 1978–2012. Int. J. Cancer.

[4] Liu C, Wu J, Chang Z (2021). Trends and age-period-cohort effects on the prevalence, incidence and mortality of hepatocellular carcinoma from 2008 to 2017 in Tianjin, China. Int. J. Environ. Res. Public Health.

[5] Zhang CH, Cheng Y, Zhang S, Fan J, Gao Q (2022). Changing epidemiology of hepatocellular carcinoma in Asia. Liver Int..

[6] Ishihara A (2021). Preoperative risk assessment for delirium after hepatic resection in the elderly: A prospective multicenter study. J. Gastrointest. Surg..

[7] Lin X, Cao Y, Liu X, Yu K, Miao H, Li T (2022). The hotspots and publication trends in postoperative delirium: A bibliometric analysis from 2000 to 2020. Front. Aging Neurosci..

[8] Jin Z, Hu J, Ma D (2020). Postoperative delirium: Perioperative assessment, risk reduction, and management. Br. J. Anaesth..

[9] Park EA, Kim MY (2019). Postoperative delirium is associated with negative outcomes and long-term mortality in elderly Koreans: A retrospective observational study. Medicina (Kaunas).

[10] Tsukakoshi D (2023). Association between postoperative delirium and heart rate variability in the intensive care unit and readmissions and mortality in elderly patients with cardiovascular surgery. Heart Vessels.

[11] Shi Z (2019). Postoperative delirium is associated with long-term decline in activities of daily living. Anesthesiology.

[12] Markar SR, Smith IA, Karthikesalingam A, Low DE (2013). The clinical and economic costs of delirium after surgical resection for esophageal malignancy. Ann. Surg..

[13] Janssen TL (2020). Long-term outcomes of major abdominal surgery and postoperative delirium after multimodal prehabilitation of older patients. Surg. Today.

[14] Burton JK (2021). Non-pharmacological interventions for preventing delirium in hospitalised non-ICU patients. Cochrane Database Syst. Rev..

[15] Berian JR (2018). Postoperative delirium as a target for surgical quality improvement. Ann. Surg..

[16] Hughes CG (2020). American society for enhanced recovery and perioperative quality initiative joint consensus statement on postoperative delirium prevention. Anesth. Analg..

[17] American Geriatrics Society abstracted clinical practice guideline for postoperative delirium in older adults. *J. Am. Geriatr. Soc.* **63**, 142-150 (2015). 10.1111/jgs.13281 PMC5901697 25495432

[18] Aldecoa C (2017). European Society of Anaesthesiology evidence-based and consensus-based guideline on postoperative delirium. Eur. J. Anaesthesiol..

[19] Xiang D, Xing H, Zhu Y (2022). A predictive nomogram model for postoperative delirium in elderly patients following laparoscopic surgery for gynecologic cancers. Support Care Cancer.

[20] Chen J, Ji X, Xing H (2022). Risk factors and a nomogram model for postoperative delirium in elderly gastric cancer patients after laparoscopic gastrectomy. World J. Surg. Oncol..

[21] Mosk CA, van Vugt J, de Jonge H, Witjes CD, Buettner S, Ijzermans JN, van der Laan L (2018). Low skeletal muscle mass as a risk factor for postoperative delirium in elderly patients undergoing colorectal cancer surgery. Clin. Interv. Aging.

[22] Choi NY, Kim EH, Baek CH, Sohn I, Yeon S, Chung MK (2017). Development of a nomogram for predicting the probability of postoperative delirium in patients undergoing free flap reconstruction for head and neck cancer. Eur. J. Surg. Oncol..

[23] Inouye SK, van Dyck CH, Alessi CA, Balkin S, Siegal AP, Horwitz RI (1990). Clarifying confusion: The confusion assessment method. A new method for detection of delirium. Ann. Intern. Med..

[24] Smulter N, Lingehall HC, Gustafson Y, Olofsson B, Engström KG (2015). Validation of the confusion assessment method in detecting postoperative delirium in cardiac surgery patients. Am. J. Crit. Care.

[25] Kumar V, Bishayee K, Park S, Lee U, Kim J (2023). Oxidative stress in cerebrovascular disease and associated diseases. Front. Endocrinol. (Lausanne).

[26] Kang HS (2023). An elevated likelihood of stroke, ischemic heart disease, or heart failure in individuals with gout: a longitudinal follow-up study utilizing the National Health Information database in Korea. Front. Endocrinol. (Lausanne).

[27] Hong CM, Galvagno SM (2013). Patients with chronic pulmonary disease. Med. Clin. North Am..

[28] Wang J (2023). Bibliometric and visual analysis of intraoperative hypotension from 2004 to 2022. Front. Cardiovasc. Med..

[29] Nomi T (2023). Effect of laparoscopic liver resection on postoperative delirium in elderly patients with hepatocellular carcinoma. J. Hepatobiliary Pancreat. Sci..

[30] Yoshimura Y (2004). Risk factors for postoperative delirium after liver resection for hepatocellular carcinoma. World J. Surg..

[31] Chen YL (2015). Low hemoglobin level is associated with the development of delirium after hepatectomy for hepatocellular carcinoma patients. PLoS One.

[32] Takeuchi M (2012). Incidence and risk factors of postoperative delirium in patients with esophageal cancer. Ann. Surg. Oncol..

[33] Kunz JV, Spies CD, Bichmann A, Sieg M, Mueller A (2020). Postoperative anaemia might be a risk factor for postoperative delirium and prolonged hospital stay: A secondary analysis of a prospective cohort study. PLoS One.

[34] Kolk A, Schwarzer C, Wolff KD, Grill F, Weingart J (2022). Factors associated with postoperative delirium in patients undergoing complex head and neck flap surgery. J. Oral Maxillofac. Surg..

[35] Kang T, Park SY, Lee JH, Lee SH, Park JH, Kim SK, Suh SW (2020). Incidence & risk factors of postoperative delirium after spinal surgery in older patients. Sci. Rep..

[36] Liu Y, Shen W, Tian Z (2023). Using machine learning algorithms to predict high-risk factors for postoperative delirium in elderly patients. Clin. Interv. Aging.

[37] Xu Y, Meng Y, Qian X, Wu H, Liu Y, Ji P, Chen H (2022). Prediction model for delirium in patients with cardiovascular surgery: Development and validation. J. Cardiothorac. Surg..

[38] Gold BT, Brown CA, Hakun JG, Shaw LM, Trojanowski JQ, Smith CD (2017). Clinically silent Alzheimer's and vascular pathologies influence brain networks supporting executive function in healthy older adults. Neurobiol. Aging.

[39] Pendlebury ST, Thomson RJ, Welch S, Rothwell PM (2022). Cognitive predictors of delirium on long-term follow-up after TIA and stroke: Population-based cohort study. Cerebrovasc. Dis..

[40] Liu F, Huang J, Hei G, Wu R, Liu Z (2020). Effects of sulforaphane on cognitive function in patients with frontal brain damage: Study protocol for a randomised controlled trial. BMJ Open.

[41] Zhou Q (2021). Predictors of postoperative delirium in elderly patients following total hip and knee arthroplasty: A systematic review and meta-analysis. BMC Musculoskelet. Disord..

[42] Honda S (2018). Risk factors for postoperative delirium after gastrectomy in gastric cancer patients. World J. Surg..

[43] Hiraki M (2021). A clinical risk analysis of early post-operative delirium after laparoscopic colorectal cancer surgery in elderly patients: A retrospective study. Int. J. Colorectal Dis..

[44] Mayhew D, Mendonca V, Murthy B (2019). A review of ASA physical status—Historical perspectives and modern developments. Anaesthesia.

[45] Ueno M, Hayami S, Tani M, Kawai M, Hirono S, Yamaue H (2014). Recent trends in hepatectomy for elderly patients with hepatocellular carcinoma. Surg. Today.

[46] Takagi K (2016). Sarcopenia and American Society of anesthesiologists physical status in the assessment of outcomes of hepatocellular carcinoma patients undergoing hepatectomy. Acta Med. Okayama.

[47] Ng K (2021). Development and validation of a novel nomogram predicting 10-year actual survival after curative hepatectomy for hepatocellular carcinoma. Surgeon.

[48] Zhang X (2019). Predictive nomogram for postoperative delirium in elderly patients with a hip fracture. Injury.

[49] Malik AT, Quatman CE, Phieffer LS, Ly TV, Khan SN (2019). Incidence, risk factors and clinical impact of postoperative delirium following open reduction and internal fixation (ORIF) for hip fractures: An analysis of 7859 patients from the ACS-NSQIP hip fracture procedure targeted database. Eur. J. Orthop. Surg. Traumatol..

[50] Vacas S, Grogan T, Cheng D, Hofer I (2022). Risk factor stratification for postoperative delirium: A retrospective database study. Medicine (Baltimore).

[51] Sánchez Acedo P, Eguaras Córdoba I, Zazpe Ripa C, Herrera Cabezón J, Tarifa Castilla A (2020). Prospective study of factors associated with postoperative delirium after urgent abdominal surgery. Cir. Esp. (Engl. Ed.).

[52] Liu J, Li J, Gao D, Wang J, Liu M, Yu D (2023). High ASA physical status and low serum uric acid to creatinine ratio are independent risk factors for postoperative delirium among older adults undergoing urinary calculi surgery. Clin. Interv. Aging.

[53] Zhu Y, Wang G, Liu S, Zhou S, Lian Y, Zhang C, Yang W (2017). Risk factors for postoperative delirium in patients undergoing major head and neck cancer surgery: A meta-analysis. Jpn. J. Clin. Oncol..

[54] Venkatakrishnaiah NK, Anandkumar UM, Wooly S, Rajkamal G, Gadiyar HB, Janakiraman P (2022). Identification of factors contributing to the development of postoperative delirium in geriatric patients with hip fractures- A prospective study. J. Family Med. Prim. Care.

[55] Okawa Y (2021). The assessment of risk factors for postoperative delirium using cubic spline curves in gastroenterological surgery. Surg. Today.

[56] Li GH, Zhao L, Lu Y, Wang W, Ma T, Zhang YX, Zhang H (2021). Development and validation of a risk score for predicting postoperative delirium after major abdominal surgery by incorporating preoperative risk factors and surgical Apgar score. J. Clin. Anesth..

[57] Kim H, Chung S, Joo YH, Lee JS (2016). The major risk factors for delirium in a clinical setting. Neuropsychiatr. Dis. Treat..

[58] Llewellyn DJ, Langa KM, Friedland RP, Lang IA (2010). Serum albumin concentration and cognitive impairment. Curr. Alzheimer Res..

[59] Oh ES (2015). Preoperative risk factors for postoperative delirium following hip fracture repair: A systematic review. Int. J. Geriatr. Psychiatry.

[60] Beloosesky Y, Hendel D, Weiss A, Hershkovitz A, Grinblat J, Pirotsky A, Barak V (2007). Cytokines and C-reactive protein production in hip-fracture-operated elderly patients. J. Gerontol. A Biol. Sci. Med. Sci..

[61] Lin S (2019). Surgical outcomes of hand-assisted laparoscopic liver resection vs. open liver resection: A retrospective propensity score-matched cohort study. Chin. J. Cancer Res..

[62] Hołówko, W. *et al.* Early adoption of laparoscopic liver surgery in Poland: A national retrospective cohort study. *Int. J. Surg.* (2023). 10.1097/JS9.0000000000000840 PMC10793755 37816169

[63] Yang Q (2023). A retrospective analysis of the incidence of postoperative delirium and the importance of database selection for its definition. BMC Psychiatry.

[64] Liu Q, Li L, Wei J, Xie Y (2023). Correlation and influencing factors of preoperative anxiety, postoperative pain, and delirium in elderly patients undergoing gastrointestinal cancer surgery. BMC Anesthesiol..

[65] Ishibashi H (2022). Postoperative delirium in lung cancer anatomical resection-analysis of risk factors and prognosis. World J. Surg..

[66] Aitken SJ, Blyth FM, Naganathan V (2017). Incidence, prognostic factors and impact of postoperative delirium after major vascular surgery: A meta-analysis and systematic review. Vasc. Med..

[67] Mou Q (2023). Preoperative anxiety as an independent predictor of postoperative delirium in older patients undergoing elective surgery for lumbar disc herniation. Aging Clin. Exp. Res..

[68] Pakrad F, Pakrad E, Darvishi N, Poorolajal J (2020). Preoperative anxiety and depression increases the incidence of delirium after coronary artery bypass graft surgery. J. Perianesth. Nurs..

